# Novel Materials for Sound-Absorbing Applications

**DOI:** 10.3390/ma19040740

**Published:** 2026-02-14

**Authors:** Eulalia Gliścińska

**Affiliations:** Textile Institute, Faculty of Textiles and Design, Lodz University of Technology, Zeromskiego Street, No. 116, 90-924 Lodz, Poland; klata@p.lodz.pl

Various places and situations are increasingly cause discomfort to humans due to the presence of disturbing sounds. The frequency of these sounds, depending on their origin, can widely vary, from low to high and from narrow to wide ranges [1]. Similarly, various possibilities and requirements exist for using materials to dampen unwanted sounds. Considering these various factors, new sound-absorbing materials with the required characteristics are being developed [2,3,4,5,6,7,8,9,10]. This Special Issue (SI), “Novel Materials for Sound-Absorbing Applications,” comprises eight publications that advance the field of novel materials for sound-absorbing applications. This Special Issues includes the latest results from experimental, theoretical, and simulation studies.

Efforts are currently directed toward reducing and managing material waste. When designing new materials, their ease of processing and reuse must be considered [11]. In line with the principles of a circular economy, new materials should be produced using recycled materials. Therefore, research has focused on developing novel sound-absorbing materials whose components are sourced from recycled materials [12,13,14]. Technologies for such materials need to be developed, and the relationship of their sound absorption properties with the structure and composition of the materials must be studied [5,6,15,16,17]. Recyclate with a high degree of fragmentation poses a particular challenge, which hinders the ability to control the structure of the sound-absorbing material [13]. Badida et al. [18] designed a test insert for a measurement system, enabling the study of granular layered structures and comparatively analyzed the acoustic properties of rubber granulate from recycled car tires used in loose and compressed form. The test results indicated that the transmission losses were higher for more compressed and thicker samples composed of finer granulate fractions. Broda et al. [19] proposed using wool waste from tanneries as a natural, environmentally friendly material to produce carpet backing, which is the most versatile material for reducing noise in an enclosed space. This nonwoven backing offers an acoustic alternative to polyester nonwoven backing and helps reduce plastic pollution. Łach et al. [20] analyzed the influence of particle size and thermal treatment of diatomite on the use of diatomite as a component enhancing the sound absorption of composites. The authors descried the characteristics of diatomite, a mineral material obtained from Polish deposits, and a method for producing a green composite from natural components: flax fibers as reinforcement, polylactide fibers as the matrix material, and diatomite powder. The findings showed that sound absorption was increased without needing to calcinate diatomite and that the finest diatomite fraction should be used.

Designing sound-absorbing materials for reducing low-frequency or broadband sound remains a considerable challenge for engineers [8,13,21,22,23]. The presented studies focused on developing novel acoustic absorber structures, including those with a controllable resonance frequency and a tunable acoustic absorption bandwidth, demonstrating progress in the design of tunable acoustic absorbers. Zhang et al. [24] presented a novel solution for reducing broadband noise. They proposed a membrane metamaterial that was shunted using digitally controlled piezoelectric patches, which ensured high sound absorption at assumed programmable frequencies. Wang et al. [25] proposed replacing the micro-gap with a bent slit in a thin, 3.1 cm thick acoustic absorber, without changing the external dimensions. This replacement significantly increased low-frequency sound absorption and resulted in a broadened frequency band. This absorber effectively achieved adjustable resonance absorption and adjustable acoustic absorption bandwidth.

The development of new sound-absorbing materials with complex structures, such as foam, powder, honeycomb, or hybrids, necessitates the development of innovative tools for designing materials and methodologies for estimating and determining sound-absorbing properties [7,26,27,28,29,30,31,32,33]. In a theoretical analysis of the acoustic properties of lightweight powders, Sakamoto et al. [34] modeled a powder layer as a longitudinally oscillating one-dimensional beam and derived a transfer matrix. The proposed model requires only three experimentally determined parameters instead of nine, as in the commonly used Biot model [35]. Gai et al. [36] constructed an improved version of the honeycomb layered structure, a Helmholtz-type acoustic metamaterial, where each honeycomb is replaced with a small Helmholtz resonator. The authors developed theoretical and finite element models for this structure. The experimental studies confirmed that this solution ensures the material is light in weight and provides improved acoustic performance. Foam materials have structures that are geometrically difficult to model because of their irregularity. Sakamoto et al. [37] developed a method for estimating the sound absorption of foam materials with the membrane removed using computed tomography images.

The timely management of post-consumer and post-industrial waste, primarily from fibrous materials (e.g., carpets, disposable products, clothing, and technical products), paper, plastic, rubber, and natural waste, is urgently needed [13,14,16,38,39,40]. Future research should focus on developing new environmentally friendly sound-absorbing materials, i.e., recycled and/or recyclable materials [41]. This often requires the use of waste materials with varying degrees of fragmentation and that has various forms. Future work should therefore address the impact of the structure of sound-absorbing material containing shredded waste on the sound absorption performance. Despite the many existing solutions, enabling individuals to isolate from noise indoors or outdoors remains a major challenge. Future research should focus on the structural development of new sound-absorbing materials, i.e., those that meet indoor safety requirements or are suitable for outdoor use in air or water. These materials should have certain properties, e.g., constituting an additional thermal insulation or electromagnetic radiation barrier; being thin, i.e., less than 1 cm, but with high mechanical strength and resistance to various external factors; absorbing high amounts of sound across the widest possible frequency range; and reusing acoustic energy [16,42,43,44,45].
